# Association between serum AMH levels and IVF/ICSI outcomes in patients with polycystic ovary syndrome: a systematic review and meta-analysis

**DOI:** 10.1186/s12958-023-01153-y

**Published:** 2023-10-23

**Authors:** Tianyi Yuwen, Ziyi Yang, Guhao Cai, Gengchen Feng, Qichen Liu, Huijia Fu

**Affiliations:** 1 The First Clinical College of Chongqing Medical University, Chongqing, 401331 China; 2 Center for Reproductive Medicine, Shandong University, Jinan, 250012 Shandong Province China; 3 School of Medicine, Jiangsu University, Zhenjiang, 212013 Jiangsu Province China; 4 The Second Clinical College of Chongqing Medical University, Chongqing, 401331 China; 5 College of Pediatrics, Chongqing Medical University, Chongqing, 401331 China; 6 Reproductive Medicine Center, The First Affiliated Hospital of Chongqing Medical University, No. 1 Youyi Road, Yuzhong District, Chongqing, 400016 China

**Keywords:** Polycystic ovary syndrome, Anti-Müllerian hormone, In vitro fertilization, Intracytoplasmic sperm injection, Assisted reproductive techniques, Systematic review, Meta-analysis

## Abstract

**Context:**

Anti-Müllerian hormone (AMH) levels are increased in polycystic ovary syndrome (PCOS) patients and are associated with PCOS severity.

**Objective:**

To evaluate the associations between serum AMH levels and in vitro fertilization (IVF)/ intracytoplasmic sperm injection (ICSI) outcomes in patients with PCOS.

**Data sources:**

PubMed, Embase, and the Cochrane Library were searched on 11 July 2022.

**Study selection:**

Studies reporting the association between serum AMH levels and IVF/ICSI outcomes in PCOS patients were considered for inclusion. The primary outcomes were clinical pregnancy, live birth, and ovarian hyperstimulation syndrome.

**Data extraction:**

Data were extracted using a standardized data extraction form. Study quality was assessed independently by two groups of researchers.

**Data synthesis:**

Nineteen studies were included in this review. Meta-analyses demonstrated that PCOS patients with a serum AMH level within the 75-100^th^ percentile had a decreased odds of clinical pregnancy (OR: 0.77, 95% CI: 0.63–0.93) and livebirth (OR: 0.71; 95% CI: 0.58–0.87) compared to those within the 0-25^th^ percentile. An increased AMH level was also correlated with an increased number of oocytes retrieved (SMD: 0.90, 95% CI: 0.30–1.51) and a lower odds of fertilization (OR: 0.92, 95% CI: 0.87–0.98). There was no significant difference in the number of MII oocytes (SMD: 1.85, 95% CI: -1.07–4.78), E_2_ on the day of hCG (SMD: 0.12; 95% CI: -0.98–1.23), or implantation (OR: 0.82, 95% CI: 0.28–2.39) between the two groups. In addition, we found significant dose–response associations between serum AMH level and clinical pregnancy, live birth, number of oocytes retrieved, and fertilization in PCOS patients.

**Conclusion:**

AMH may have clinical utility in counseling regarding IVF/ICSI outcomes among women with PCOS who wish to undergo fertility treatment. More large-scale, high-quality cohort studies are needed to confirm these findings.

## Introduction

Polycystic ovary syndrome (PCOS) is characterized by hyperandrogenism, chronic anovulation, and polycystic ovaries, with various reproductive and metabolic sequelae [1]. It continues to be one of the most prevalent endocrine conditions among women of reproductive age, the leading cause of anovulatory infertility, and a significant risk factor for type 2 diabetes and mental health issues [2]. Studies have demonstrated how PCOS affects fertility and pregnancy [3, 4]. Women with PCOS have increased risks of adverse maternal and neonatal outcomes [5]. Assisted reproductive technology (ART), such as in vitro fertilization (IVF) or intracytoplasmic sperm injection (ICSI), can provide effective treatment options for infertility in women with PCOS [6].

Anti-Müllerian hormone (AMH) is secreted solely by the granulosa cells of preantral and small antral follicles [7]. Known for its low intracycle and intercycle variability, the AMH level is a significantly more accurate and reliable measure of ovarian reserve than the antral follicle count (AFC) or FSH concentration, and this has led to its adoption by clinicians in the counseling of women regarding their reproductive lifespans and the impact of gonadotoxic chemotherapy, radiotherapy or surgery on the ovarian reserve [8]. Serum AMH levels are significantly higher in women with PCOS than in those with normal ovulatory function [9]. This observation has led to the hypothesis that AMH could be a valuable surrogate marker for the diagnosis of PCOS and prediction of ART outcomes. However, while it is well established that AMH is correlated with ovarian response and is a good predictor of oocyte yield following ART, it is still controversial whether it may also be associated with qualitative outcomes of ART [10, 11]. Moreover, uncertainty exists as to whether increased prepregnancy AMH levels affect the ART outcome of pregnancy in women with PCOS [12]. In a cohort trial on 2436 women with PCOS undergoing IVF/ICSI, researchers found that the live birth rate (LBR) and clinical pregnancy rate (CPR) of fresh embryo transfer cycles were lower with higher baseline AMH levels than with low or average AMH levels [13, 14]. In contrast, studies have reported a null association between serum AMH levels and IVF/ICSI outcomes in patients with PCOS [15, 16]. Specifically, they demonstrated that AMH may have a predictive role among non-PCOS patients but not among PCOS patients. In addition, although a lot of opposite conclusions have been reported so far, no studies have systematically analyzed and clarified the association between prepregnancy serum AMH and IVF/ICSI outcomes in PCOS patients. Therefore, we aimed to summarize currently available evidence regarding the association between serum AMH level and IVF/ICSI outcomes in PCOS patients.

## Materials and methods

This meta-analysis is registered with PROSPERO (registration number: CRD42022300037) and was conducted in accordance with the Meta-analyses of Observational Studies in Epidemiology (MOOSE) checklist and the Preferred Reporting Items for Systematic Reviews and Meta-Analyses (PRISMA) guidelines [17, 18]. No formal ethical approval was acquired for this study.

### Search strategy

Two groups of authors (TY, ZY and CG, GF) independently screened each record. PubMed, Embase, and the Cochrane library were comprehensively searched for relevant studies from the respective inceptions of these databases to 11 July 2022. Keywords including “polycystic ovary syndrome”, “in vitro fertilization”, “intracytoplasmic sperm injection”, “assisted reproductive technology”, and “anti-Müllerian hormone” and their entry terms were used in the database searches. There was no specified date, country, or language restriction. We did not include any IVF/ICSI or pregnancy outcomes in the initial search because the exact outcomes may not be present in the title or abstract but might be described in the full text instead. We also manually searched Google Scholar and examined the reference lists of all included studies and key journals in the related field to include all potentially eligible studies. After selecting studies by their titles and abstracts, the full text of potential studies was obtained and examined for eligibility.

### Inclusion and exclusion criteria

The key questions for this study were based on the Populations, Exposures, Comparison, and Outcome (PECO) framework as follows: (1) the study population was PCOS patients undergoing IVF/ICSI; (2) the exposure was a higher serum AMH level (e.g., 75-100^th^ percentile) than that of the general population of PCOS patients; (3) the comparator was a lower serum AMH level (e.g., 0-25^th^ percentile) than that of the general population of PCOS patients; and (4) the primary outcomes of interest for this review were clinical pregnancy, livebirth, and ovarian hyperstimulation syndrome (OHSS). The secondary outcomes included E_2_ on the day of hCG, number of oocytes retrieved, number of MII oocytes, implantation, fertilization, obstetric outcomes, and neonatal outcomes. Case reports, case series, reviews, comments, letters, and conference abstracts were excluded.

### Data extraction and risk of bias assessment

The following data were extracted from the included studies: study name, first author, year of publication, country, study design, participant inclusion and exclusion criteria, PCOS diagnostic criteria, measurement of AMH, number of participants in each group, and ovarian stimulation protocol. Study authors were contacted for additional information or missing data if necessary. Considering that all eligible studies had a cohort design, their quality (risk of bias) was assessed using the Newcastle‒Ottawa quality assessment scale (NOS), with a maximum score of 9 representing the highest quality. Studies rated with a score of more than 6 were rated as high quality. Both data extraction and risk of bias assessment were conducted independently by two groups of authors (TY, ZY and CG, GF), and all discrepancies were resolved by consultation and discussion with QL and HF.

### Statistical analysis

The statistical analyses were performed using Stata/SE (version 5.1), and further analysis was performed with R (version 4.1.1). In R, the meta and dmetar packages were used to obtain pooled results. The dosresmeta package was used to conduct dose–response meta-analysis. Odds ratios (ORs) were calculated for dichotomous outcomes with a 95% confidence interval (Cl), while standardized mean differences (SMDs) with 95% CIs were calculated for continuous outcomes. Heterogeneity was checked using *I*^2^ statistics. Meaningful heterogeneity was determined if the *I*^2^ was greater than 50%. In this case, a random-effects model was used to pool studies. The robustness of the results was assessed using the leave-one-out method. If a study classified their participants into a low-AMH group (the 0-25^th^ percentile), average-AMH group (the 25-75^th^ percentile), and high-AMH group (the 75-100^th^ percentile), then the high-AMH group was compared with the low-AMH group and a dose–response meta-analysis was conducted. If a study provided data based on the classification of the 75-90^th^ percentile group and 90-100^th^ percentile group, then the latter group was compared with the former group in our meta-analysis. We also calculated weighted mean AMH cutoff values for each group and displayed the results in forest plots.

## Results

### Study selection

**Fig. 1:**
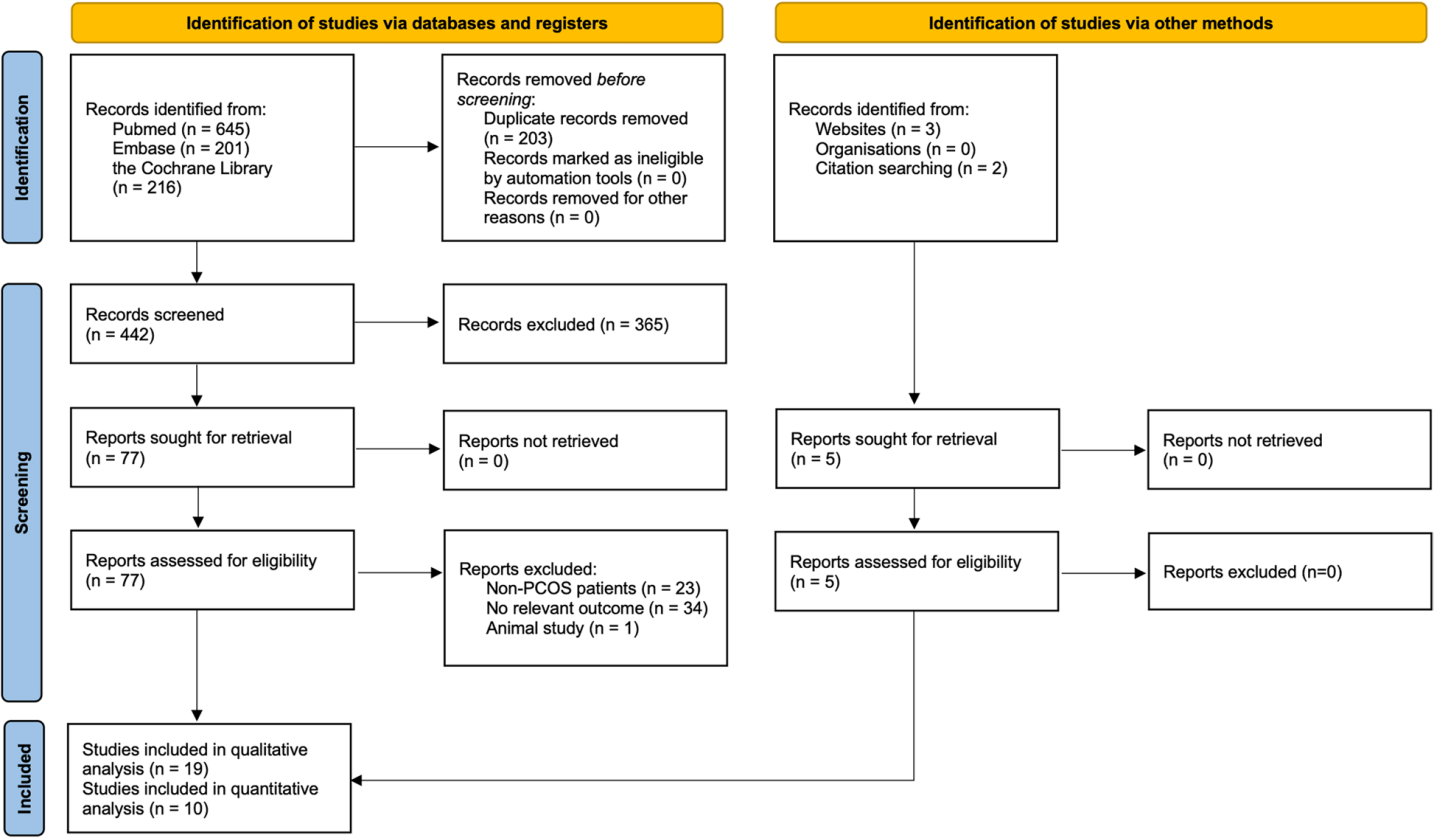
PRISMA flow diagram of the study selection process

As shown in Fig. 1, 650 studies were identified by initial databases and manual searches. A total of 203 studies were removed due to duplicate records, and 365 were excluded after assessing their titles and abstracts. The remaining 82 studies underwent full-text review, and 63 studies were not eligible for inclusion. Finally, 19 studies fulfilled the eligibility criteria and were included in the qualitative analysis, with 10 included in the quantitative analysis (meta-analysis).

### Study characteristics

Table 1 summarizes the characteristics of the 19 studies. All 19 studies were cohort studies, and the NOS score ranged from 5 to 9 (median 6). The classifications of the level of serum AMH were not consistent among these studies. Seven studies classified participants into the low-AMH group (the 0-25^th^ percentile), average-AMH group (the 25-75^th^ percentile), and high-AMH group (the 75-100^th^ percentile) [13, 15, 16, 19–22]; three studies classified participants into the 75–90^th^ percentile group and 90-100^th^ percentile group [14, 23, 24]. The remaining studies did not classify participants into different groups based on AMH levels but analyzed AMH levels as a continuous variable [22, 25–33] and thus were not included in the quantitative analysis (meta-analysis). All 19 studies used the Rotterdam criteria for the diagnosis of PCOS.

**Table 1:** Characteristics of the included studies

Authors	Year	Inclusion criteria	Number of participants	Ovarian stimulation protocol	AMH levels (ng/ml)	NOS score
Liu et al.	2022	Not mentioned	Low-AMH group (the 0-25^th^ percentile) (*n* = 746); average-AMH group (the 25th-75th percentile) (*n* = 1486); high-AMH group (the 75th-100th percentile) (*n* = 741)	Standardized luteal phase downregulation protocol with GnRH agonist protocol	25th: 2.25; 75th: 5.71	8
Tabibnejad et al.	2018	PCOS patients aged < 43 years; had fewer than three previous failed IVF/ICSI cycles; scheduled for day 3 embryo transfer	50 PCOS patients	The majority (90%) were stimulated using an antagonist protocol, and the others received an antagonist protocol (3%) or a microdose flare protocol (7%)	PCOS group mean AMH (SD): 7.67 (4.78)	5
Tal et al.	2020	PCOS patients underwent their first fresh IVF/ICSI cycles	Low-AMH group (the 0-25th percentile) (*n* = 46); average-AMH group (the 25th-75th percentile) (*n* = 92); high-AMH group (> 75th percentile) (*n* = 46)	The stimulation protocol included either pituitary downregulation via GnRH agonist in a long protocol or a GnRH antagonist to prevent premature ovulation	25th: 3.32; 75th: 8.27	5
Guo et al.	2021	PCOS patients underwent their first fresh IVF/ICSI cycles	Low-AMH group (the 0-25th percentile) (*n* = 611); average-AMH group (the 25th-75th percentile) (*n* = 1216); high-AMH group (> 75th percentile) (*n* = 609)	All enrolled patients received GnRH agonist or antagonist protocol	25th: 6.77; 75th: 14.30	6
Kaya et al.	2010	PCOS patients underwent COH in ICSI cycles	Low-AMH group (the 0-25th percentile) (21 cycles); average-AMH group (the 25th-75th percentile) (39 cycles); high-AMH group (> 75th percentage) (20 cycles)	All patients received a standard GnRH agonist protocol	25th: 2.54; 75th: 3.85	5
Arslanca et al.	2021	PCOS patients underwent FET cycles	Group 1: 75th–90th percentile (Group 1) (*n* = 66); higher than the 90th percentile (Group 2)(*n* = 44)	Not mentioned	75th: 6.12; 90th: 8.12	6
Xi et al.	2012	PCOS patients aged < 43 years; underwent first IVF cycle	Low-AMH group (the 0-25th percentile) (*n* = 41); average-AMH group (the 25th-75th percentile) (*n* = 82); high-AMH group (> 75th percentile) (*n* = 41)	Long downregulation protocol	25th: 4.85; 75th: 8.82	7
Du et al.	2021	PCOS patients underwent IVF cycles	The patients were divided into two groups: Group A (*n* = 90): The AMH serum levels in group A patients were less than 6.99 ng/ml Group B (*n* = 110): The AMH serum levels in group B patients were greater or equal to 6.99 ng/ml	Not mentioned	25th: 2.71; 75th: 6.45	9
Ho et al.	2018	PCOS patients aged 18–42 years; underwent IVM	Not mentioned	Women received injections of FSH 100 IU/day on cycle days 3, 4 and 5 and were given an injection of hCG 10,000 IU on cycle day 6. Oocyte pick-up (OPU) was scheduled for 36 h later	Mean AMH (SD) for all participants: 12.3 (3.6)	6
Hu et al.	2018	Women who undergo IVF treatment and fresh embryo transfer	75-90th percentile (*n* = 117) and 90-100th percentile (*n* = 41)	Ovarian stimulation was achieved using recombinant FSH or human menopausal gonadotrophin with various flexible protocols	75th: 4.97; 90th: 7.99	6
Du et al.	2021	PCOS women who undergo IVF/ICSI treatment, fresh embryo transfer and singleton delivery	BMI < 24; AMH levels: < 2.71 ng/ml (*n* = 333), 2.71–4.08 ng/ml (*n* = 330), 4.09–6.45 ng/ml (*n* = 351), > 6.45 ng/ml (*n* = 325). BMI ≥ 24; AMH levels: < 2.71 ng/ml, 2.71–4.08 ng/ml, 4.09–6.45 ng/ml, > 6.45 ng/ml	All patients received a standard GnRH agonist protocol	Cutoff value: 6.99	6
Sahmay et al.	2013	< 40 years of age, FSH < 15 mIU/mL, had normal PRL and TSH levels, no previous history of ovarian surgery	Low-AMH group (the 0-25th percentile) (*n* = 36); average-AMH group (the 25th-75th percentile) (*n* = 77); high-AMH group (the 75th-100th percentile) (*n* = 37)	All women underwent gonadotropin-releasing hormone (GnRHa) agonist	25th: 4.23; 75th: 8.66	7
Kamel et al.	2016	Aged 20–39 years, undergoing IVF/ICSI cycles	The patients were divided into two groups. Group A (*n* = 524): AMH < 4.6 ng/ml Group B (*n* = 452): AMH > 4.6 ng/ml	All women underwent the gonadotropin-releasing hormone (GnRH) antagonist (GnRH-ant) protocol	Cutoff value: 4.60	6
Muharam et al.	2022	Aged 24–41 years, underwent an IVF cycle with (1) serum AMH level > 4 ng/ml and (2) underwent the GnRH antagonist protocol	Not mentioned	All patients received a standard GnRH agonist protocol	PCOS mean AMH (SD): 7.59 (4.61)	5
Acharya et al.	2022	Aged < 44 years with an AMH level of ≥ 5 ng/m, undergoing their first fresh autologous IVF cycles	Not mentioned	Not mentioned	Median AMH (IQR) of all participants: 7.1 (5.8–9.5)	7
Arabzadeh et al.	2010	Aged 21–37 years with BMI between 17 and 32	Control group (*n* = 42); PCOS (*n* = 26)	All women underwent gonadotropin-releasing hormone (GnRH) antagonist (GnRH-ant) protocol	Not mentioned	5
Hsu et al.	2018	Not mentioned	Term Deliveries (*n* = 392); Preterm Deliveries (*n* = 40)	Not mentioned	Not mentioned	6
Chen et al.	2017	Patients with PCOS, fallopian tube problems without PCOS, or treatment due to male infertility without PCOS	PCOS (*n* = 59); Control (*n* = 120)	Agonist protocol (*n* = 30); Antagonist protocol (*n* = 29)	PCOS mean AMH (SD): 11.86 (4.79)	5
Guan et al.	2022	Women (≥ 35 years) with PCOS and women with tubal factor infertility, underwent their first fresh cycles and subsequent frozen cycles	PCOS (*n* = 106); Tubal infertility group (*n* = 1073)	The protocol used for controlled ovarian stimulation (COS) was a long gonadotrophin-releasing hormone (GnRH)-agonist protocol for IVF or ICSI	PCOS mean AMH (SD): 45.07 (24.02) (pmol/L)	6

### Primary outcomes

#### Clinical pregnancy

**Fig. 2:**
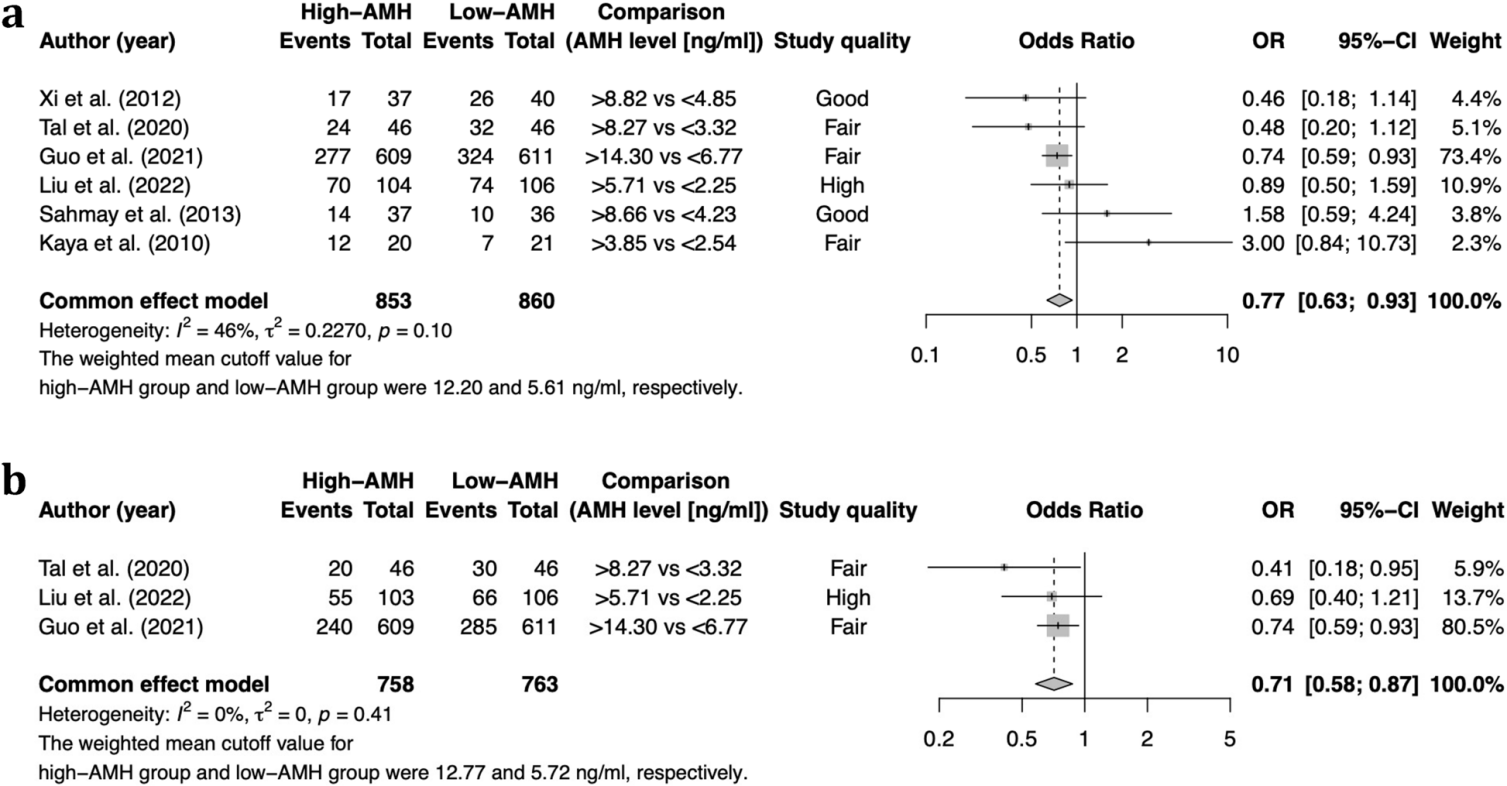
Meta-analysis results for primary outcomes. **A** Forest plot for clinical pregnancy rate. **B** Forest plot for live birth rate

Seven studies analyzed clinical pregnancy; six stratified their patients into a low-AMH group (the 0-25^th^ percentile), average-AMH group (the 25-75^th^ percentile), and high-AMH group (the 75-100^th^ percentile) [13, 15, 16, 19–21]. The overall clinical pregnancy rate was 48.5% (414/853) and 55.0% (473/860) in the high-AMH group and the low-AMH group, respectively. Meta-analysis demonstrated that the odds of a clinical pregnancy were significantly lower if patients were classified in the high-AMH group (OR: 0.77, 95% CI: 0.64–0.93) (Fig. 2). There was a moderate degree of heterogeneity (*I*^2^ = 46%). In addition, Du et al. [25] examined clinical pregnancy rate in a cohort of 200 PCOS patients aged 25 to 36 years undergoing IVF-ET. Patients were divided into two different groups based on a cutoff AMH level of 6.99 ng/L. Their results showed that clinical pregnancy rate was significantly lower if AMH was greater than 6.99 ng/L (*p* = 0.001). The result of the dose–response meta-analysis is shown in Fig. 4a. We observed an inverse linear association between prepregnancy serum AMH level and clinical pregnancy in PCOS patients (*p* = 0.008). The heterogeneity was not significant (I^2^ = 44.4%). The OR (95% CI) of clinical pregnancy was 0.996 (0.994, 0.999) per 1% increase in the AMH percentile.

#### Live birth

Seven studies analyzed live birth; three studies stratified their patients into a low-AMH group (the 0-25^th^ percentile), average-AMH group (the 25-75^th^ percentile), and high-AMH group (the 75-100^th^ percentile) [13, 15, 20]. The incidence of live birth (per treatment cycle) was 49.9% (381/763) in the low-AMH group and 48.9% (315/643) in the high-AMH group. Meta-analysis demonstrated that the odds of a live birth were significantly lower if patients were classified into the high-AMH group than if they were classified into the low-AMH group (OR: 0.71; 95% CI: 0.58–0.87) (Fig. 2). Heterogeneity was not detected (*I*^2^ = 0%). The result of the dose–response meta-analysis is shown in Fig. 4b. We observed an inverse linear association between prepregnancy serum AMH level and live birth in PCOS patients (*p* < 0.001). The heterogeneity was not significant (I^2^ = 0%). The OR (95% CI) of live birth was 0.995 (0.993, 0.998) per 1% increase in the AMH percentile.

This section contains a summary of findings that cannot be meta-analyzed. Ho et al. examined live birth rate in a cohort of 921 women with PCOS who underwent IVM priming with hCG [27]. While high AMH levels do indicate a high number of oocytes and a high oocyte maturation rate, univariate analysis did not reveal an association between AMH level and live birth after the transfer of the first embryo after IVM (OR: 1.02; 95% CI: 0.98–1.06). Notably, this result may be applicable only to the specific IVM technique used in this study, namely, the transfer of day-2 embryos, which is not a regular practice at many IVF centers. Tabibnejad et al. [26] investigated the relationship between serum AMH levels and ICSI outcomes in 50 PCOS patients with 289 embryos. In this scenario, their findings suggested that AMH was not an accurate predictor of a live birth (AUC = 0.59 [95% CI, 0.42–0.76]). However, among women with tubal factor infertility, AMH had a moderate predictive value for a live birth (AUC = 0.70 [95% CI, 0.55 to 0.85]). Guan et al. [28] analyzed the cumulative live birth rate in 160 PCOS patients of advanced age (≥ 35 years). All patients underwent their first fresh cycles and subsequent frozen cycles within one year. Their results demonstrated that patients with an AMH level above 32.12 pmol/L were likely to have a 72% (HR, 1.72; 95% CI, 1.08–2.73, *p* = 0.023) and 34% (HR, 1.34; 95% CI, 1.07–1.68, *p* = 0.010) improvement in cumulative live birth rate compared to those with AMH levels below 7.85 pmol/L and 7.85–32.12 pmol/L, respectively. Acharya et al. [32] divided their patients based on an AMH cutoff level of 12 ng/ml. Their results showed that AMH was negatively associated with live birth (OR, 0.93; 95% CI, 0.90–0.96) up to an AMH level of 12 ng/ml. Beyond 12 ng/ml, the association was attenuated (OR, 1.01; 95% CI, 0.99–1.04).

#### Ovarian hyperstimulation syndrome

Three studies analyzed the incidence of OHSS. Tal et al. [15] reported a retrospective cohort study in a sample of 184 women with PCOS who underwent their first fresh IVF/ICSI cycles. Women were stratified into 3 groups according to the 0-25^th^ (< 3.32 ng/ml), 25-75^th^ (3.32–8.27 ng/ml), or 75-100^th^ (> 8.27 ng/ml) percentile of serum AMH concentration. The stimulation protocol included either pituitary downregulation via GnRH agonist in a long protocol or a GnRH antagonist to prevent premature ovulation. No difference regarding the OHSS incidence was found among the three groups. When Kamel et al. [33] divided patients into two groups (AMH cutoff value: 4.6 ng/ml) and used GnRH antagonist, they found that the incidence of severe OHSS was significantly higher in patients with AMH > 4.6 ng/ml (*p* = 0.026). In addition, Muharam et al. [30] tried to determine the cutoff value of AMH to predict hyperresponse in PCOS patients undergoing controlled ovarian stimulation. The AUC of the ROC curve was 0.626 (95% CI; sensitivity: 71.8%; specificity: 52.7%), indicating poor predictive quality.

### Secondary outcomes

#### E_2_ on day of hCG

**Fig. 3:**
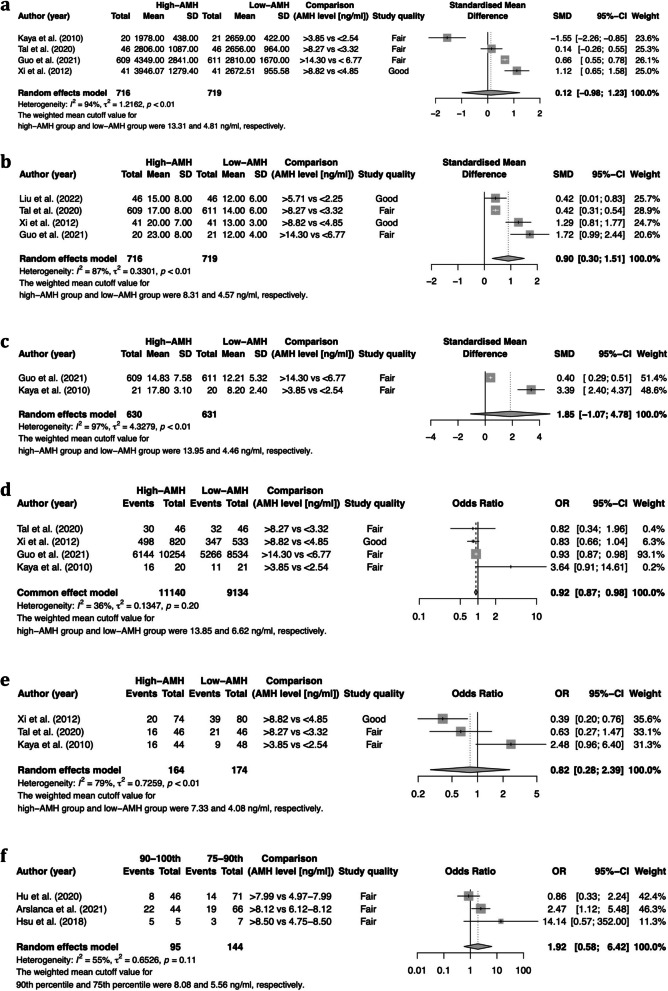
Meta-analysis results for secondary outcomes. **A** Forest plot for E_2_ on hCG day, **B** forest plot for number of oocytes retrieved, **C** forest plot for number of MII oocytes, **D** forest plot for fertilization rate, **E** forest plot for implantation rate, and **F** forest plot for preterm birth

**Fig. 4:**
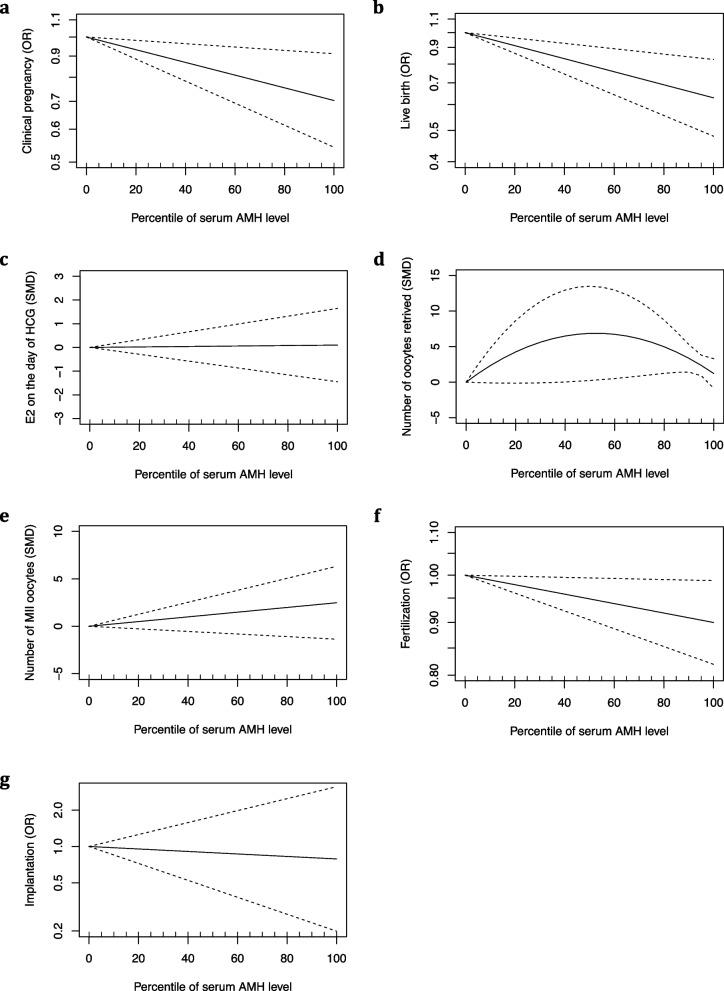
Dose–response meta-analysis results for **A** Clinical pregnancy rate, **B** Live birth rate, **C** E_2_ on hCG day, **D** Number of oocytes retrieved, **E** Number of MII oocytes, **F** Fertilization rate, **G** Implantation rate

Four studies analyzed E_2_ on the day of hCG and stratified their patients into a low-AMH group (the 0-25^th^ percentile), average-AMH group (the 25-75^th^ percentile), and high-AMH group (the 75-100^th^ percentile) [13, 15, 16, 21]. The pooled results found a null association between serum AMH levels and E_2_ on the day of hCG (SMD: 0.12; 95% CI: -0.98–1.23) (Fig. 3a). Sensitivity analysis demonstrated that the study by Kaya et al. [21] affected the robustness of the meta-analysis. After excluding this study, the difference in E_2_ on the day of hCG became significant (SMD: 0.63; 95% CI: 0.13–1.14). The result of the dose–response meta-analysis is shown in Fig. 4c. Using a linear model, we did not observe a dose–response association between prepregnancy serum AMH level and E_2_ on the day of hCG in PCOS patients (*p* = 0.901).

### Number of oocytes retrieved

Eight studies analyzed the number of oocytes retrieved: four stratified their patients into a low-AMH group (the 0-25^th^ percentile), an average-AMH group (the 25-75^th^ percentile), and a high-AMH group (the 75-100^th^ percentile) [13, 15, 16, 21]. The meta-analysis showed a significantly increased number of oocytes retrieved in women with PCOS who were classified into the high-AMH group compared with the low-AMH group (SMD: 0.90, 95% CI: 0.30–1.51) in a random-effects model. There was a high degree of heterogeneity (*I*^2^ = 87%) (Fig. 3b). The result of the dose–response meta-analysis is shown in Fig. 4d. Using a quadratic model, we observed a nonlinear inverted U-shaped association between prepregnancy serum AMH level and the number of oocytes retrieved in PCOS patients (*p* = 0.002). PCOS patients with an AMH level between the 39^th^ percentile and the 97^th^ percentile had a significantly increased number of oocytes retrieved. The heterogeneity was significant (I^2^ = 99.1%).

This section contains a summary of findings that cannot be meta-analyzed. Ho et al. [27], using the study design described earlier in this paper, reported that AMH showed a significant positive correlation with the number of oocytes by univariate analysis (coefficient = 0.28; *p* = 0.001). Similarly, Tabibnejad et al. [26] prospectively evaluated the number of oocytes retrieved in a cohort of 50 PCOS patients undergoing ICSI. They also found a positive correlation (Spearman’s r = 0.45, *p* = 0.001). However, when using multivariable analysis in a sample of 59 PCOS patients, Chen et al. [29] found a null association (r = -0.059, *p* = 0.685). Arslanca et al. [14] reported the same outcome in a cohort of 110 PCOS patients who underwent FET. Patients were categorized into the AMH 75-90^th^ percentile group (*n* = 66) and 90-100^th^ percentile group (*n* = 44), and no significant differences in terms of the number of oocytes retrieved were noted between the two groups.

### Number of MII oocytes

Three studies analyzed the number of MII oocytes. Two stratified their patients into a low-AMH group (the 0-25^th^ percentile), an average-AMH group (the 25-75^th^ percentile), and a high-AMH group (the 75-100^th^ percentile) [13, 21]. Although there was a trend toward a greater number of MII oocytes in the high-AMH group (SMD: 1.85, 95% CI: -1.07–4.78), the difference did not reach significance in a random-effects model. There was a high degree of heterogeneity (*I*^2^ = 97%) (Fig. 3c). In addition, Tabibnejad et al. [26] reported a significant positive correlation between AMH concentration and the number of MII oocytes (Spearman r = 0.42, *p* = 0.002). The result of the dose–response meta-analysis is shown in Fig. 4e. Using a linear model, we did not observe a dose–response association between prepregnancy serum AMH level and the number of MII oocytes in PCOS patients (*p* = 0.206).

### Fertilization

Six studies reported fertilization rate, and four of those studies stratified their patients into a low-AMH group (the 0-25^th^ percentile), average-AMH group (the 25-75^th^ percentile), and high-AMH group (the 75-100^th^ percentile) [13, 15, 16, 21]. The incidence of overall fertilization (per treatment cycle) was 61.9% (5656/9134) in the low-AMH group and 60.0% (6688/11140) in the high-AMH group. The meta-analysis demonstrated significantly decreased odds of fertilization in women with PCOS whose AMH was classified in the high-AMH group in a fixed-effects model (OR 0.92, 95% CI 0.87–0.98) (Fig. 3d). There was a moderate degree of heterogeneity (*I*^2^ = 36.1%). In addition, Du et al. [25] and Arabzadeh et al. [31] reported a null association between AMH and the fertilization rate (*p* > 0.05). The result of the dose–response meta-analysis is shown in Fig. 4f. We observed an inverse linear association between prepregnancy serum AMH level and fertilization in PCOS patients (*p* = 0.027). The heterogeneity was not significant (I^2^ = 44%). The OR (95% CI) of fertilization was 0.999 (0.998, 1.000) per 1% increase in the AMH percentile.

### Implantation

Five studies analyzed implantation rate, and three of those studies stratified their patients into a low-AMH group (the 0-25^th^ percentile), average-AMH group (the 25-75^th^ percentile), and high-AMH group (the 75-100^th^ percentile) [15, 16, 21]. The overall incidence of implantation was 39.7% (69/174) in the low-AMH group and 31.7% (52/164) in the high-AMH group. The meta-analysis found no association between AMH level and implantation rate (OR: 0.82, 95% CI: 0.28–2.39) (Fig. 3e). There was a high degree of heterogeneity (*I*^2^ = 79%). Du et al. [25] reported that the incidence of implantation was 41.1% (37/90) in the low-AMH group (< 6.99 ng/L) and 20.91% (23/110) in the high-AMH group (> 6.99 ng/L). Additionally, Arabzadeh et al. [31] reported that AMH was not associated with implantation rate in women with PCOS (r = -0.299, *p* = 0.138) but was positively associated with implantation rate in the non-PCOS control group (r = 0.305, *p* = 0.05). The result of the dose–response meta-analysis is shown in Fig. 4g. We did not observe a dose–response association between prepregnancy serum AMH level and implantation in PCOS patients (*p* = 0.735).

### Cycle cancellation

Two studies analyzed cycle cancellation. Acharya et al. [32] stratified their patients with an AMH cutoff value of 12 ng/ml. In both groups, an increasing AMH level was associated with a higher probability of cycle cancellation (OR: 1.12, 95% CI: 1.10–1.15 and OR: 1.03, 95% CI: 1.01–1.05 in the AMH < 12 ng/ml group and > 12 ng/ml group, respectively). In the analysis of the reasons for cycle cancellation, the authors reported that in the AMH < 12 ng/ml group, each 1-unit increase in AMH level was associated with an 11% increase in the odds of embryo transfer cancellation because of the OHSS risk (OR, 1.11; 95% CI, 1.07–1.16). Xi et al. [16] stratified their 164 participants into 3 groups according to the < 25^th^ (< 4.85 ng/ml), 25^th^ to 75^th^ (4.85–8.82 ng/ml), or > 75^th^ (> 8.82 ng/ml) percentile of serum AMH concentration. Embryo transfers cancelled due to OHSS risk from the low-, middle-, and high-serum AMH groups were 1, 4 and 7 cases, respectively.

### Obstetric outcomes

Three studies analyzed miscarriages. Liu et al. [20] examined this outcome in a cohort of 2973 infertile women, including 418 women with PCOS undergoing their first IVF treatments. The incidence of miscarriage was 8.1% (6/74) in the low-AMH group, 19.1% (29/152) in the average-AMH group, and 17.1% (12/70) in the high-AMH group. Although there were more high-quality embryos transferred in the average-AMH group than in the low-AMH group, the difference in miscarriage rate was not significant among these three groups, indicating that AMH was not associated with miscarriage rate among PCOS patients. Du et al. [25] also reported the early miscarriage rate in their two subgroups. The findings suggested that the rate of early miscarriage was significantly lower among the participants in the low-AMH group, with an incidence of early miscarriage of 6.67% (6/90) in the low-AMH group and 19.09% (21/110) in the high-AMH group (*p* < 0.001). Notably, this study investigated only the early miscarriage rate of patients, while the patient’s late pregnancy process was not studied.

GDM was reported in two studies [14, 25]. In the comparison of the GDM incidence between the serum AMH 75-90^th^ percentile group and the 90-100^th^ percentile group, there was no association between AMH and GDM. However, Du et al. [25] suggested that patients with AMH greater than 6.99 ng/ml had an increased incidence of GDM (*p* < 0.001). In addition, preeclampsia and PPROM were reported by only one study [14], with no association found.

### Neonatal outcomes

Four studies reported preterm birth [14, 22–24]. Meta-analysis was performed to compare the preterm birth rate between the 75-90^th^ percentile group and the 90-100^th^ percentile group (AMH) (Fig. 3f), and a null association was found (OR: 1.92, 95% CI: 0.58–6.42). Meanwhile, Du et al. [22] reported that a higher AMH (75-100^th^ percentile) was associated with an increased risk for preterm birth among women with a BMI ≥ 24 kg/m^2^ (OR: 2.10, 95% CI: 1.01–4.37) but not among women with a BMI < 24 kg/m^2^ (OR: 0.78, 95% CI: 0.35–1.73) after adjusting for multiple confounding factors, including maternal age, BMI, duration of infertility, and basal antral follicle count. This study also selected “small for gestational age”, “large for gestation age”, “low birth weight”, and “macrosomia” as outcomes of interest. In brief, no significant differences were found in the rates of these outcomes among patients in the different serum AMH groups (adjusted OR ranging from 0.91 to 1.13).

## Discussion

This paper summarizes currently available evidence concerning the association between prepregnancy serum levels of AMH and IVF/ICSI outcomes among women with PCOS and substantially strengthens the theory that a higher level of AMH is associated with a subsequently lower clinical pregnancy rate and live birth rate. The findings presented here could reveal a more significant role for AMH in women with PCOS in clinical settings, and they represent a step toward more precise medicine by demonstrating the value of AMH in the analysis of the risk of adverse ART outcomes in an individual with PCOS.

Regarding the primary outcomes, our results demonstrated that serum AMH levels were negatively associated with clinical pregnancy rate and live birth rate in PCOS patients undergoing IVF/ICSI. Notably, these results were in contrast with the findings of prior studies based on the general population, which demonstrated that a higher AMH level is associated with a higher live birth rate and live birth rate. A previous meta-analysis [34] showed that the pooled diagnostic OR for AMH as a predictor of clinical pregnancy rate among 4324 women in the general population with unspecified ovarian reserve was 2.10, whereas the area under the curve (AUC) of the summary receiver operation characteristic (ROC) curve was 0.634. Thus, the role of AMH in predicting IVF/ICSI outcomes among PCOS patients is different from that in the general population. PCOS is characterized by elevated AMH levels, which are due to both the increased number of small antral follicles that express AMH the most and the overexpression of AMH and anti-Mullerian hormone receptor type 2 by their granulosa cells (GCs) [2, 35, 36]. In GCs from women with PCOS, AMH expression is upregulated by high levels of luteinizing hormone (LH), androgens, and androgen receptors. Studies have also found a positive correlation between AMH levels and PCOS severity [37, 38]. In severe PCOS, although patients do have a higher number of follicles, follicle development is suppressed, which may result in a higher number of oocytes retrieved but no increase in MII oocytes [39]. In women, AMH inhibits the recruitment of primordial follicles out of the resting oocyte pool and may suppress FSH actions, contributing to ovulatory disturbances. This is consistent with our study, as we found that patients with AMH levels in the 75-100^th^ percentile range had an increased number of total oocytes but not MII oocytes. Next, following oocyte retrieval and during fertilization and implantation, indicators such as fertilization rate and implantation rate may also be similar regardless of AMH levels due to obesity, insulin resistance, poor luteal function, and poor endometrial receptivity [40, 41]. In the present study, when women with AMH levels within the 75-100^th^ percentile and those with AMH levels within the 0-25^th^ percentile were compared, the former had a decreased fertilization rate and an implantation rate comparable to that of the latter group. The ORs were 0.92 and 0.82, respectively. In the general population, researchers have found that patients with low AMH levels had a higher rate of MII oocytes [42]. This may be associated with the number of follicles that grow in the ovary. Compared with a large quantity of oocytes, a few oocytes may obtain more sufficient nutrition from the ovary to support their maturation. Additionally, increased levels of AMH cleavage have been found to be related to various metabolic parameters in both control women and women with PCOS, which has a negative impact on implantation and endometrial receptivity. Overall, these factors led to the observed lower clinical pregnancy rate and live birth rate in high-AMH PCOS patients.

PCOS patients are more likely to suffer from OHSS due to a higher sensitivity and exaggerated response to ovarian stimulation protocols, particularly COS with gonadotropins [43]. Severe OHSS can lead to serious complications, including pleural effusion, acute renal insufficiency, and venous thromboembolism, and it can even be life-threatening. Therefore, every attempt should be made to identify patients who are at the highest risk for OHSS. The present study analyzed the association between AMH and the risk of OHSS in PCOS patients and found inconsistencies in the results of prior studies. In general, AMH has a poor predictive quality in OHSS. In addition, two studies demonstrated increasing cycle cancellation events due to OHSS risk in patients with elevated AMH levels. With similar results, in a retrospective cohort study of 134 general women with elevated AMH levels (> 5 ng/ml), women with AMH > 10 ng/ml had significantly higher rates (> threefold) of OHSS [44]. In another study, AMH levels in women with OHSS were sixfold higher than those in age- and weight-matched controls [45]. Notably, available studies were very limited, and patients received different ovarian stimulation protocols, which made it difficult to generalize the results. Clinical guidelines have demonstrated that AMH values > 3.4 may be useful to predict increased OHSS risk, but this cutoff point needs further validation [46]. Overall, AMH may be useful for planning ovarian stimulation protocols and counseling patients regarding risk. However, these measures should be used with caution since clear cutoff points have not been validated in the literature.

There are some limitations to this study. First, many of the included studies did not report adjusted effect estimates, which are less biased by confounders compared to crude estimates. Second, heterogeneity was observed in part of our meta-analysis. Current evidence is hampered by the differences in basic demographic characteristics, such as age and BMI, ovulation stimulation protocols, and AMH assays. Finally, we did not discuss other pregnancy outcomes, such as the multiple pregnancy rate or cesarean delivery, in our meta-analysis due to scarce information in the current literature.

In conclusion, this study assessed currently available evidence on the association between serum AMH levels and IVF/ICSI outcomes in PCOS patients. Our results suggest that an increased serum AMH level is inversely associated with clinical pregnancy, live birth, and fertilization; a higher serum AMH level is also associated with a higher number of oocytes retrieved, though comparable number of MII oocytes, in women with PCOS undergoing ART. Thus, AMH may be a useful risk stratification tool for PCOS women undergoing IVF/ICSI and may have clinical utility in counseling regarding IVF/ICSI outcomes among women with PCOS who wish to undergo fertility treatment. More large-scale, high-quality cohort studies are needed to confirm these findings.

## Data Availability

Original data generated and analyzed during this study are included in this published article or in the data repositories listed in References.
